# Supplementary material for: MSTN Regulatory Network in Mongolian Horse Muscle Satellite Cells Revealed with miRNA Interference Technologies
Source: Genes (Basel). 2022 Oct 11;13(10):1836. doi: 10.3390/genes13101836 (PMC9601437; doi:10.3390/genes13101836)
Supplement: Supplementary file 1 [file genes-13-01836-s001.zip › genes-1889472-supplementary.pdf]

**Table S1. Q-PCR primers for DEG validation**

| Gene ID | Forward 5'-3' | Reverse 5'-3' |
|---|---|---|
| MSTN | GAACCCAGGCGCTGGTATTT | CTCTGGGGTTTGCTTGGTGT |
| MYOG | GGCGTGCAAGGTGTGTAAGA | ATGGTTTCATCCGGGAAGGC |
| MYH14 | GCTGCAACCTGTTTGTGCTGAA | GGGAACTTGCAGGGCTGT |
| MYL9 | TTCCTTCACTCGCGCCCAA | TGGAGGTCCCCAAAGCTACG |
| MYOD1 | GACGGCTCTCTCTGCAACTT | AGGTGCCATCGTAGCAGTTC |
| MYH9 | GAGCCGTATGGAAACCCGAG | GAGCAAAGTTTCCTCCGAGGC |
| CDKN1C | GTGACTGGATTGCCTGCGAC | ACCGTGTTCTCAGGGTTGGA |
| CCND2 | GCCCTTCATGGTGGTGAATGATG | GCTTGCGCTTGAATTCTGCG |
| CDK3 | CAGCCAGACCTCTCTGCCT | CACCACGCAGGAAGTCCAG |
| CCNB2 | CTGTTCGAGTCGTCGCACATC | GTAGAGGTAGGGCAGGATCTGG |
| CCNB3 | TTGGCAGTAAAGCTGGTGGA | TTGGCAGTAAAGCTGGTGGA |
| WNT5A | GTGAATTCCCGAGGAGGGAC | GTGAATTCCCGAGGAGGGAC |
| DNER | AGATTCGGCAAGATGCTGCT | GGCCATCTATGAGGGAGCAC |
| PAX5 | AGAAAAGGAGTCCGACGACAAG | AGATCCTCAGAGTCCACCACAC |
